# ‘I'm institutionalised … there's not much I can do’: Lived experience of housing related delayed discharge

**DOI:** 10.1111/1440-1630.12821

**Published:** 2022-05-25

**Authors:** Christina Pui Ting Chuah, Anne Honey, Karen Arblaster

**Affiliations:** ^1^ The University of Sydney Camperdown New South Wales; ^2^ Nepean Blue Mountains Local Health District Penrith New South Wales Australia

## Abstract

**Introduction:**

Delayed discharge from inpatient mental health units is the continued hospitalisation of consumers after being assessed as ready to be discharged. This is common in adult inpatient mental health services and is usually due to a lack of appropriate housing for discharge. Research indicates a range of possible negative impacts, but no studies have explored consumers' perspectives on this issue. This study explores consumers' perspectives of the experience of housing related delayed discharge (HRDD).

**Method:**

Using a grounded theory approach, in‐depth interviews were conducted with 10 consumers. All consumers were experiencing HRDD from inpatient mental health units in one Sydney local health district. The data were analysed using constant comparative analysis.

**Findings:**

A lack of choice and control was the central theme that characterised participants' experiences. The combined experience of being delayed in hospital and being homeless led to a lack of choice and control over the basics in life, how consumers spent their time and with whom, and their futures. This lack of autonomy was shaped by the features of the hospital and participants' personal circumstance. The hospital features included rules and routines, physical and social environments, resources, and support from staff. Personal circumstances included individual situations, social networks, and support from community organisations. Participants described a variety of impacts, including reduced mental and physical well‐being, and anticipated difficulty transitioning back into the community.

**Conclusion:**

This study is the first of its kind and provides consumer perspectives on the impacts of HRDD on their well‐being and recovery. The inability to participate in meaningful and necessary occupations is an occupational injustice and implies the need for occupational therapists to advocate for both the prevention of HRDD through government investment in affordable and readily available housing and the mitigation of its effects through modified hospital environments and practices.

**Key Points for Occupational Therapy**

- HRDD is characterised by a lack of choice and control over one's daily life.
- HRDD is an infringement of consumers' occupational rights.
- Occupational therapists should advocate for all consumers who experience HRDD.

## 1 INTRODUCTION

Delayed discharge from inpatient mental health units is the continued hospitalisation of people living with mental health challenges after being assessed as medically fit for discharge (Poole et al., 2014). It is a significant issue in Australia and internationally and, in inpatient adult mental health, is almost universally caused by housing related difficulties. This includes lack of housing options, housing affordability issues, and difficulty attaining housing supports and services (Gaughan et al., 2017; Nguyen et al., 2022; Poole et al., 2014). This is referred to as housing related delayed discharge (HRDD). A recent Australian study estimated that HRDD could cost the Australian health system more than $269.5 million annually, making it an expensive problem (Nguyen et al., 2022).

More importantly, HRDD represents a contravention of the rights of people living with mental health challenges (hereafter referred to as ‘consumers’, a commonly accepted term in Australia that highlights the importance of autonomy and choice for people living with mental health challenges) (NSW Mental Health Commission, 2014). The United Nations Convention on the Rights of Persons with Disabilities (CRPD) (United Nations, 2006) states that signatory countries have a responsibility to uphold the right to liberty of people living with a disability. In countries like Australia, where most mental health acute units are secure and coercive practices are commonly employed, people's freedom of movement and autonomy is severely restricted, infringing on this right to liberty. Further, hospitalisation can cause difficulties in managing housing payments, placing consumers who had a home prior to their hospitalisation at an increased risk of losing their accommodation (Mental Health Council of Australia, 2009).

The human rights issue of delayed discharge is of particular concern to occupational therapists because of its potential impacts on occupational participation. The World Federation of Occupational Therapists (World Federation of Occupational Therapy (WFOT), 2006) advocates that everyone has a right to participate in occupations that promote satisfaction, freedom, and well‐being. However, hospitalisation and lack of appropriate housing may prevent and exclude consumers from engaging in occupations that are personally meaningful and promote health and well‐being (Akther et al., 2019; Foye et al., 2020; Honey et al., 2017). Occupational therapists play two primary roles in relation to these concerns in contemporary Australian mental health inpatient units. First, they work to enable participation in meaningful occupations within the setting (McKay et al., 2008). Second, they conduct detailed assessment, planning, report writing and preparation of referrals, and applications for housing and funding for supports. These latter processes are lengthy, complex, and time consuming and, due to lack of time and resources, are commonly prioritised over more therapeutically focused activities to minimise delays to discharge (Nguyen et al., 2022).

There is a growing literature exploring delayed discharge from mental health inpatient units. However, it is mainly focused on identifying rates and predictors of delayed discharge and discussing potential impacts on the hospital systems (Everall et al., 2019; Impey & Milner, 2013; Little et al., 2019; Poole et al., 2014). Predictors of delayed discharge include having various medical and psychiatric comorbidities, lower socioeconomic status, and high levels of drug and alcohol abuse (Little et al., 2019). Impacts on hospital systems include blocking beds needed for other consumers; increased expenditure due to bed usage by consumers fit for discharge; and increased staff burnout due to the additional workload and stress surrounding delayed discharge (Gaughan et al., 2017; Nguyen et al., 2022).

A limited number of studies explore mental health consumers' experiences of prolonged admissions (Akther et al., 2019; Chevalier et al., 2018; Everall et al., 2019; Haynes et al., 2011). These suggest that the impacts are considerable and include: deterioration in mental and physical health; loss of dignity, power, and autonomy; feelings of restriction and scrutiny; limited access to meaningful activities; the sense of time standing still; hopelessness and boredom; and alienation from family and friends. The effects are compounded by the coercive nature of mental health care and restrictive ward routines, which lead to distress, fear, and powerlessness (Akther et al., 2019). These research papers consider the effects of prolonged hopsitalisation and coercive environments but do not focus on the specific effects of HRDD. HRDD may have similar impacts to those listed above, given that it extends hospital stay. However, different or additional impacts may result from the additional contexts of homelessness and being clinically assessed as ready for discharge. Furthermore, most research to date has been conducted outside of Australia. While delayed discharge is seen as problematic internationally, different research methods and definitions of delayed discharge make comparison between countries difficult (Little et al., 2019; Nguyen et al., 2022; Poole et al., 2014) and different mental health and social care systems are likely to influence people's experiences. For example, Australia has a comparatively high rate of involuntary hospitalisation compared to other Organisation for Economic Co‐operation and Development countries (Sheridan Rains et al., 2019).

Occupational therapists should play a vital role in advocating and promoting participation in desired occupations that positively influence well‐being (Hammell & Iwama, 2012). To more effectively address the threat of HRDD to occupational participation and therefore reduce harms to consumers, it is important for occupational therapists to understand the lived experiences of those directly affected. Taking into account consumers' experience and perspectives in designing and delivering mental health services is increasingly valued by government and health‐care services as they move towards adopting a more recovery‐oriented approach (Department of Health, 2017). Therefore, this study addresses the following research question: How do Australian consumers experience HRDD from an inpatient mental health unit?

## 2 METHODS

### 2.1 Study design

The study adopted a constructivist grounded theory approach. This was appropriate due to the limited literature on the topic of HRDD and, more specifically, consumers' experience of HRDD (Charmaz, 2014; Chun Tie et al., 2019). A constructivist approach accounted for the subjectivity of participants' experiences and researchers' interpretation of the data (Charmaz, 2014). Due to being a small‐scale exploratory study, a cyclical process of theoretical sampling was not possible. As such, the study loosely followed a grounded theory approach that used the key grounded theory techniques of simultaneous data collection and analysis, memo‐writing, a two‐step coding process, and constant comparative analysis (Charmaz, 2014). Ethical approval was granted by the Nepean Blue Mountains Local Health District (NBMLHD) Human Research Ethics Committee (Ref: REGIS2019/ETHO1294).

### 2.2 Study setting

The study was conducted in a Local Health District (LHD) on the outskirts of Sydney in New South Wales (NSW), Australia. The LHD has a large tertiary referral hospital with four inpatient mental health units and several smaller, geographically dispersed hospitals, one of which includes a 15‐bed general acute unit. Wards are locked, so consumers' freedom of movement is severely restricted regardless of whether they have been admitted voluntarily or involuntarily. With approval from the treating team, consumers can usually access leave to complete tasks such as banking, shopping, or spending time with family.

Much of the occupational therapists' time is occupied with assessments and discharge planning including preparing detailed rehabilitation referrals, National Disability Insurance Scheme (NDIS), and other applications for housing and support services and advocating for consumers as they navigate complex discharge arrangements. Other tasks include attending multiple multidisciplinary ward rounds, case conferences, working with consumers' families, and supporting consumers to access other essential supports. Along with nursing and diversional therapy staff, occupational therapists also run programed activities. However, as they work office hours only, implementation of evening and weekend activities depends on the availability of nursing staff to supervise. While there is also some opportunity for individualised therapeutic interventions, the primary focus for occupational therapists is discharge planning to facilitate timely discharge and support patient flow.

### 2.3 Recruitment

Participants were recruited from two of the inpatient units between February and September 2020. They were a purposive sample of inpatients who met the following criteria: (a) working age (16–64 years old) and (b) assessed by their treating team as ready for discharge but awaiting housing. Consumers awaiting aged‐care placement were excluded as their experiences of delayed discharge were likely to be different (Impey & Milner, 2013). Criterion sampling was the purposive sampling method used; everyone who met the inclusion criteria was invited to participate (Chun Tie et al., 2019). To avoid perceived coercion, potential participants were identified and informed about the study by their occupational therapist or social worker who, it was made clear, was not involved with the study. Consumers who were interested in participating gave permission for the university researchers to contact them to explain the study. The third author, allied health manager at the LHD, did not know who participated or have access to identifiable data. Participants were provided with a plain language participant information sheet, and all provided written‐informed consent.

After eight interviews, no new themes were arising, and the categories were well filled out, indicating that data saturation had been achieved. Therefore, participant recruitment was terminated after 10 interviews.

### 2.4 Data collection

Data were collected using semi‐structured, in‐depth interviews (Minichiello et al., 2008). Based on previous literature and contextual knowledge, an interview guide containing broad questions was developed in collaboration with a mental health clinician and peer support worker. At the beginning of the interview, participants were asked for demographic data such as their age, mental health diagnosis, and estimated length of housing‐related delayed discharge. Thereafter, the guide was used to elicit detailed responses about participants' experiences of HRDD. For example, participants were asked to describe their daily routine prior to and during hospitalisation and how they felt they were affected by being in hospital after their treating team had designated them ready for discharge. The flexibility of the guide allowed discussion and generation of new concepts during the interviews (Minichiello et al., 2008). Interviews were conducted by the first or second author and lasted between 20 and 60 min. Nine interviews were conducted in person, within hospital wards, and one was conducted by telephone. Interviews were transcribed verbatim. Data were analysed prior to subsequent interviews. This process of simultaneous data collection and analysis allowed authors to modify the interview guide, enabled continuous refinement of emerging codes and concepts, and allowed authors to ascertain when data saturation occurred (Chun Tie et al., 2019).

### 2.5 Data analysis

Codes were developed inductively to fit the data. Data from interviews were coded using constant comparative analysis, a systematic process of line by line coding and category development involving both substantive and theoretical coding (Charmaz, 2014). Substantive coding involved line‐by‐line analysis that generated codes for a phrase, statement, or section. For example, the statement ‘there's limited things we can do’ was initially coded as ‘lack of things to do in hospital’. As new substantive codes were created, they were compared to existing codes to find similarities, differences, and gaps in knowledge. Similar codes were combined to form higher level codes. For example, ‘lack of things to do in hospital’ became part of the broader code, ‘lack of choice about how I spend my time’. As substantive codes became clear, links between them were developed. This is known as theoretical coding and supported the development of the study's conceptual framework (Charmaz, 2014). For example, it was identified that the extent of consumers' choices was shaped by specific physical and social features of the hospital environment.

To enhance rigour, the first and second author independently coded and discussed the coding of the first two interviews. For subsequent interviews, the first author coded the data and discussed emerging concepts with the second author. A qualitative research software package, NVivo, was used to manage and organise the data. A summary of findings and invitation to comment was sent to nine participants who consented to this. None provided additional feedback. Researchers sought advice from a peer support worker when interpreting the findings. Based on the data, a conceptual framework was developed to explain consumers' experience of HRDD.

## 3 FINDINGS

### 3.1 Participants

Table 1 provides details of participants' self‐reported characteristics. All were given pseudonyms.

**TABLE 1:** Participant information (self‐reported; *n* = 10)

Gender	
Male	5
Female	5
Age of participants	
Mean	39
Range	22–61
Length of hospital stay	
<1 month	2
1–12 months	4
>12 months	1
Unsure	3
Estimated length of HRDD	
<1 month	4
1–2 months	2
>2 months	1
Unsure	3
Intended discharge destination	
Own home to be purchased or rented	3
Temporary accommodation (e.g., motel)	3
Department of housing	1
Group home	2
Unsure	1
Diagnosis	
Schizophrenia and other psychotic disorders	8
Bipolar and related disorders	4
Depressive disorders	3
Participants who identified more than 1 mental health diagnosis	6
Other diagnoses (e.g., autism spectrum disorder, attention deficit hyperactivity disorder, and cognitive impairments)	3
Physical conditions (e.g., diabetes and asthma)	3
Time since diagnosis	
<1 year	2
1–10 years	2
>10 years	3
Unsure	3

### 3.2 Conceptual framework

**FIGURE 1:**
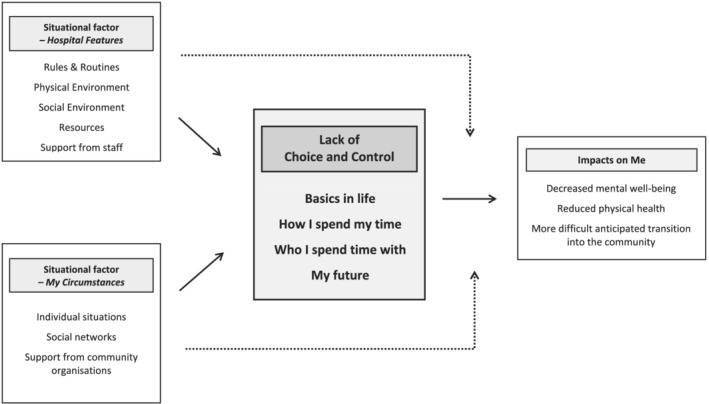
Conceptual framework of participants' experience of HRDD

Figure 1 depicts the conceptual framework developed from the data. Participants' experiences of HRDD were characterised by a lack of choice and control, the central category in this framework. This lack of choice and control led to various impacts on consumers and was influenced by two situational factors.

It is important to note that most participants did not distinguish between a time when they needed and did not need to be in hospital, with several disputing whether their hospitalisation was needed at all. Participants did not describe a specific experience of ‘housing‐related delayed discharge’ but, rather, a combined experience of extended hospitalisation and homelessness. For brevity, we refer to the combined situation as HRDD but acknowledge that this is our own conceptualisation.

In our findings, the central category—lack of choice and control—is described first and in most detail. The impacts of a lack of choice and control are then outlined, and the situational factors that influence the central category are summarised.

### 3.3 Lack of choice and control

The central experience of HRDD for all participants was lack of choice and control. As Michael noted, ‘I'm institutionalised … there's not much I can do. Whereas when I'm outside I can do what I like, when I want’. HRDD was characterised by lack of choice and control over four areas of life: (a) basics in life, (b) how I spend my time, (c) who I spend time with, and (d) my future.

#### 3.3.1 Basics in life

Participants spoke of being unable to exercise choice and control over basic occupations like eating, sleeping, taking medication, and performing self‐care. This affected their mental and physical health and well‐being. Participants were ‘forced to do things at certain times …, have a shower at this time, eat at this time’ (Gabriel). For example, James noted, ‘there's set times that we get the food … does not help you when you are hungry’, while Mary reported being unable to participate in daily self‐care activities when desired: ‘if I want a razor, I've got to go see them … I cannot just do whatever I want, like what I need to do’.

Crowded and noisy hospital wards, coupled with regular monitoring and surveillance by hospital staff, meant that participants had little control over when they slept. Frequent ‘room searches’ where ‘nurses come in with torches every hour or so’ (Michael) were intrusive and affected participants sleep: ‘sleeping is the worst. Everyone knows you do not get much sleep in the hospital. But in a mental health ward, it's worse’ (John).

Hospital rules left little room for self‐management of medication. For example, to limit the side effects, Mary noted, ‘I never used to really take the medication in the morning, I'd take just a small dose to keep my levels’. However, while in hospital, participants had to take the medication they were given when it was given, even if they did not understand its purpose.

#### 3.3.2 How I spend my time

All 10 participants noted that the biggest effect of being in hospital was the inability to use their time for meaningful occupations. Although participants noted that activities, like exercise, art, and crafts, were offered in hospital, they were not always available. Michael reported, ‘there's a gym, but the nurses have to watch you … so if the nurses are not available, you cannot use the weights’. Participants often could not watch the television program of their choice because ‘other people in here take control of the television’ (Eve). Even reading the newspaper depended on ‘if there's a free nurse available to go to the hospital kiosk to purchase those newspapers’ (Gabriel).

Furthermore, the resources and activities offered in hospital did not necessarily suit participants' needs and interest. Gabriel noted, ‘there's things like painting and drawing …, but we are not kids, there's no kids in here, we are all adults’. Hospital resources were sometimes old and unreliable, with Eve reporting ‘the computer stuffs [up] all the time’. Engaging in other meaningful occupations, such as surfing, shopping, and caring for family, was usually impossible.

#### 3.3.3 Who I spend time with

Being in locked wards with restricted leave meant that participants were unable to choose who they spent time with. Participants often noted being ‘forced to live with’ unfamiliar people: ‘there's no escape, you are stuck all together, there's no sort of time out’ (Gabriel). The constant admission and discharge of other consumers also meant that the hospital ‘environment is changing every single day’ and that ‘it'll never get to a good state’ (Gabriel). While ‘pleasant’ interactions with staff and consumers were occasionally reported, several participants reported witnessing frequent screaming, arguing, and threatening behaviour: ‘it's always loud, there's always someone going off, I do not feel like, at home’ (Michael).

HRDD also meant that participants were unable to choose when and where they could see their loved ones. For several, seeing their friends and spending time with family were activities they most looked forward to once discharged.

#### 3.3.4 My future

Participants lacked choice and control over important aspects of their futures, such as when they would be discharged and where they would live. Restrictions around hospital leave and internet access limited participants' ability to do the occupations needed to arrange their accommodation: ‘we only have an hour here or there, … we cannot make appointments [with agents]’ (Gabriel). This difficulty in organising accommodation from hospital and the limited available housing options meant that participants had to rely on hospital staff, housing services, or family to arrange their accommodation, further limiting their ability to choose where to live.

As such, several participants anticipated that they would have to compromise on their discharge location and be discharged to non‐preferred accommodation. James, for example, agreed to accept a place in a group home, despite his reluctance, while four participants noted they would accept temporary accommodation, hoping this would give them a chance to look for suitable permanent housing.

Four participants also reported that HRDD restricted their ability to participate in other occupations that they needed or wanted to do to help them move their lives forward, such as finding employment, enrolling in Technical and Further Education, and resolving legal issues.

### 3.4 Impacts on me

Participants noted three key impacts that resulted from a lack of choice and control: reduced mental well‐being; decreased physical health; and a more difficult anticipated transition back into the community.

All 10 participants reported having reduced *mental well‐being*, most often due to being unable to engage in desired occupations. Many reported frustration and boredom: ‘I'm more agitative … agitated and irritable while I'm in here, yeah, because I cannot do the things I want’ (Mary). The central importance of choice and control was highlighted by two participants who expressed frustration over their lack of autonomy even though they reported that their daily routines while hospitalised were not appreciably different when they were outside of hospital. For example, John reported that he would usually ‘just walk back and forth drinking coffee [and] eating food’ whether in hospital or at home. Most participants described feeling lonely and ‘cut‐off’ (Eve) from their loved ones and many noted that living in crowded and ‘claustrophobic’ hospital wards, with unfamiliar people, was often ‘stressful’, anxiety provoking, and destabilising for their mental health.

Several participants noted that being in hospital for so long affected their *physical health* challenges getting adequate nutrition, exercise, or sleep, which sometimes made them feel unwell: ‘I'm doing it a bit hard without many greens’ (Simon), and ‘I have not had much of a day for a while, because I've been … sleepy’ (Eve).

Participants also anticipated a more difficult *transition back into the community*. Mary noted, ‘when I get out, I know I'm going to have a bit more anxiety about getting around doing things’, while Gabriel explained, ‘it'll be a slower transition. It'll be slow and frustrating’. Other participants were concerned that being unable to practise or demonstrate their independent living skills in hospital might affect their transition. For example, Simon thought that his chances of getting accommodation would be affected by not having had the opportunity to show that he was ‘alright in cleaning and cooking’. However, two participants also described benefits of staying in hospital, despite the negative impacts. For example, Mary found it preferable to the alternative of being discharged into homelessness: ‘I cannot find accommodation just yet, that's why it's a good thing that I am allowed to stay here until next month’.

### 3.5 Situational factors

It is apparent in the above analysis and examples that participants' experiences of lack of choice and control were shaped by two situational factors: (a) hospital features and (b) my circumstances. These situational factors also influenced the extent to which the lack of choice and control impacted participants, as depicted by the dotted arrows in Figure 1. For instance, lacking control over who participants spent time with had a worse impact if other consumers were argumentative or aggressive or if the participant valued solitude. Because these situational factors are visible in the examples provided in the above categories, they are summarised only briefly here.

#### 3.5.1 Hospital features

Hospital features which shaped participants' choices and experiences were the rules and routines, physical and social environments, resources, and support from staff.

*Hospital rules and routines* were particularly critical in shaping participants' choices. Most prominently, being in locked wards, participants could only leave the hospital for a limited time. Often participants were only allowed to leave the unit when escorted by approved family members or service providers, either due to being held under the Mental Health Act or perceived absconding risk. The duration and conditions of leave varied between individual patients but were always determined by their treating team. They were also subject to fixed ward schedules and routine monitoring and surveillance by staff, which affected participants' sleep and well‐being. Six of the 10 interviews were also conducted during COVID‐19 hospital restrictions. Three of those participants noted that these restrictions exacerbated the restrictive features of the hospital, as for a period they were not allowed leave or visitors.

*The physical environment* influenced participants' choices about how to spend their time and with whom. This led to reduced opportunities for spending time alone, relaxing, and sleeping.

*The social environment* affected what people could do and the extent to which people were affected by not being able to choose their company. While positive social interactions provided distraction and made hospitalisation more bearable, participants' experiences were negatively affected when other consumers were noisy, argumentative, or threatening.

*Resources* like the gymnasium, television, outdoor courtyard. and computer increased options for occupation. But these resources were limited, and some were not ideal or adequate for everyone. The availability of hospital staff to provide or supervise activities was another valuable hospital resource which either enabled or limited participants' options for activities.

*Support from staff* to access housing and manage housing related issues was critical to participants' choices and experiences. Most reported receiving support from staff to find accommodation, explain the housing process, and complete paperwork. However, this was not consistent, and some experienced a lack of such support.

#### 3.5.2 My circumstances

Participants' circumstances such as individual situations, social networks, and support from community organisations affected the extent of their choice and control and influenced how the lack of autonomy affected them.

*Individual situations* included personal and contextual characteristics. All participants needed housing, but some were in a better position to acquire it. For example, some had the financial resources and knowledge of the housing market or were entitled to social housing. Participants' dispositions and relationships with other consumers and staff also impacted their experience of HRDD. For instance, Gabriel felt that his calm and tolerant disposition helped to reduce the impacts of other consumers' negative behaviours.

*Social networks*, such as support from family, influenced participants' experience of HRDD. Participants with family members who visited them, brought them food, took them out, and helped them to arrange discharge accommodation experienced more choice. However, the amount of family support varied between participants.

*Support from community organisations* was particularly important to participants who did not have family support. Service providers from non‐government organisations or community mental health would sometimes visit participants, take them for escorted leave, or help arrange discharge accommodation. They also promised support beyond hospitalisation, with participants' transition into the community.

## 4 DISCUSSION

This study is, to the best of our knowledge, the first to examine consumers' experiences of HRDD. A lack of choice and control was the central theme. This encompassed a lack of control over daily occupations and routines, including necessary occupations for finding accommodation and preparing to transition to the community. Some features of the hospital environment and personal circumstances exacerbated these experiences, while other environmental and personal factors mitigated them. Overall, the impacts on consumers were negative. These included decreased mental wellbeing and reduced physical health and anticipation of greater difficulties transitioning into the community. Interestingly, participants did not distinguish between the overall experience of the admission and the experience of the period deemed as HRDD. They often did not appear to be aware that there was a point in their admission where they were assessed by clinicians as well enough to be discharged as soon as suitable housing was sourced. However, participants did specifically identify the restrictions of the inpatient mental health environment as preventing them from completing occupations that would enable them to secure housing and facilitate their discharge. Therefore, HRDD can be considered as an extension of the period consumers experience the negative impacts of the restrictive inpatient environment and as a barrier to resolving housing concerns.

Previous research has highlighted the necessity of meaningful occupations for recovery and wellbeing (Foye et al., 2020; Marshall et al., 2020). However, a lack of meaningful activities is common in mental health inpatient settings (Akther et al., 2019; Foye et al., 2020). Like most other mental health units in Australia, the inpatient units in this study were locked, restricting freedom of movement and thereby infringing consumers' human rights (Lindgren et al., 2019). This exacerbated the loss of choice and control our participants experienced and has been described elsewhere as causing a sense of imprisonment (Chevalier et al., 2018; Lindgren et al., 2019).

The findings highlight HRDD as a pressing human rights and occupational justice issue that serves as a call to action for occupational therapists. Occupational justice requires equitable access to opportunities and resources ‘to do, be, belong and become what people have the potential to be and the absence of avoidable harm’ (Wilcock & Hocking, 2015, p. 414). The experiences of our participants demonstrate barriers to these opportunities and resources and the resulting negative impacts on their recovery and wellbeing. A limited range of occupations was available, and access to the opportunities and resources to participate in even these occupations was limited by practices such as restricting access to spaces and equipment based on staff availability to supervise. Restricted meal options negatively influenced physical health, along with limited access to exercise. While these barriers to occupational justice may be temporary and occur in relation to an inpatient admission, HRDD extends the length of time that participants experience these conditions. This can be for very long periods of time (Nguyen et al., 2022), compounding the negative impacts on health and wellbeing from which full recovery can be challenging.

Occupational therapists can address factors that perpetuate occupational injustice at microlevel (individual consumer), mesolevel (intra‐organisational) and macrolevel (population) (Bailliard et al., 2020). However, occupational therapists may not always be cognisant of occupational injustices within health‐care settings (Bailliard et al., 2020; Galvin et al., 2011). Critical reflexivity is needed to devise ways to challenge the status quo of current health‐care practices to address injustices, such as those experienced by our participants (Gerlach, 2015; Hammell, 2015). At a microlevel, occupational therapists could focus more on enabling individual participation in meaningful occupations, though they are often restricted by staffing levels and competing demands. At a mesolevel, occupational therapists could address the restrictive practices that limit opportunities and resources for occupational participation, including occupations associated with securing housing and attaining supports that will enable longer term occupational wellbeing.

At a macrolevel, HRDD contravenes consumers' rights (United Nations, 2006) to live independently, be included in the community (Article 19), and participate in all areas of life (Article 9). However, the real‐world alternative is often a discharge into homelessness as one of our participants noted. Homelessness is an infringement of a consumer's right to an adequate standard of living, including adequate housing (Article 28) and may well be the more detrimental option given the critical importance of housing for both occupational participation and mental health (Mental Health Council of Australia, 2009; Thomas et al., 2010). Thus, the root of the justice issues involved with HRDD is a lack of suitable and accessible housing for consumers.

Given that HRDD is symptomatic of widespread housing problems for people living with mental illness (Brackertz et al., 2020; Honey et al., 2017), its prevention will require increased housing options including government investment in public, community, and affordable housing. Agreements between state run housing and mental health services need to be reviewed for their effectiveness in improving housing outcomes. Increased housing support within the community and cross sector collaboration between housing and mental health service providers are also required. Such collaboration, as seen in NSW under the Housing and Accommodation Support Initiative (HASI), has been shown to reduce the number of people experiencing homelessness, lower health‐care expenditure, and improve consumers' health outcomes (Brackertz et al., 2018). HASI provides consumers with access to secure housing, clinical services, and housing support (Bruce et al., 2012). However, there is an extensive wait of up to 10 years as HASI services rely on social housing stock (Bruce et al., 2012).

In NSW, the Housing and Mental Health Agreement and Implementation Plan (Family and Community Services, 2011, 2012) articulates how social housing and state mental health services will work together to ‘improve the housing outcomes and general wellbeing of people with mental health problems and disorders who are living in social housing or who are homeless or at risk of homelessness’ (Family and Community Service, 2011, p. 6). However, neither this agreement nor the implementation of the NDIS appears to have resolved the problem of HRDD (Nguyen et al., 2022). The need for ongoing advocacy from the occupational therapy profession for improved social housing and supports for people living with mental health conditions, including more effective housing and mental health collaboration and services like HASI, is clear.

### 4.1 Limitations

The findings from this study are based on the perspectives of a small number of volunteer participants from one local health district in NSW, Australia. Therefore, as with any qualitative study, readers should assess the applicability of the findings to their own local situations. Participants' experiences of HRDD may be different from consumers experiencing HRDD who chose not to participate. Study participants were all current inpatients, and, while they could discuss their anticipated transition back into the community, we do not know what the actual transition will be like. Therefore, future studies should explore the perspectives of consumers who have transitioned back into the community after experiencing HRDD to uncover any post‐hospital impacts of HRDD. The COVID‐19 hospital restrictions, although not a prominent feature of the interviews, may have exacerbated the impacts of HRDD. Except for one category (my future), which emphasised the interaction between hospital restrictions and housing choices, most aspects of the findings may be equally applicable to people whose discharge was delayed for other, non‐housing related reasons. However, further research with this group is needed to clarify this. This paper represents only the subjective perspectives of consumers. Perspectives of other stakeholders, such as occupational therapists, other ward staff, or families, may well be different. Lastly, it is acknowledged that people living with serious mental health concerns may well experience limitations in their abilities to participate in meaningful occupations, even when living in the community. For example, Harvey et al. (2006) found that people with mental health diagnoses who live the community still experience difficulties participating in meaningful occupations even when they do not experience positive or negative symptoms. This speaks to the need for both policy and occupational therapy interventions at an individual and population level to address the occupational needs of this population more broadly.

## 5 CONCLUSION

HRDD is characterised by a lack of choice and control over one's daily life. If the Australian government is committed to upholding the rights of persons with disabilities, as per their obligations to the CRPD, and to providing recovery‐oriented mental health services, it is imperative that HRDD is prevented through investment in affordable and readily available housing and support options and that its effects are minimised through modified hospital environments and practices. Given the implications of HRDD for occupational justice, the occupational therapy profession should be at the forefront of efforts to bring about these changes.

## CONFLICT OF INTEREST

The authors declare no conflict of interest.

## AUTHOR CONTRIBUTIONS

All authors contributed to the conceptualisation and planning of the project, inspired by Author 3's observations of problems associated with HRDD in the LHD. Data were collected by Authors 1 and 2, an occupational therapy honours student and academic, respectively. All authors contributed to data analysis and the final paper.

## Data Availability

Research data are not shared due to potential identifiability of narrative data.
